# Troponin efficacy in the diagnosis of acute coronary syndrome in patients with chronic renal failure

**DOI:** 10.1097/MD.0000000000037280

**Published:** 2024-03-01

**Authors:** Ecem Ermete Güler, Umut Payza, Ahmet Kayali, Süleyman Kirik, Efe Kanter

**Affiliations:** a Emergency Medicine, Izmir Ataturk Research and Training Hospital, Izmir, Turkey; b Emergency Medicine, Izmir Katip Çelebi University, Faculty of Medicine Department, Izmir, Turkey.

**Keywords:** acute coronary syndrome, efficacy, emergency service, renal failure, troponin

## Abstract

There is no consensus on whether cardiac troponins with high reliability values should be different diagnostic criteria for acute myocardial infarction in patients with and without renal dysfunction. Although it is often emphasized that the etiology of elevated troponin levels in chronic kidney disease (CKD) remains unclear, elevated cardiac troponin (cTnT) levels have been associated with increased subclinical cardiac damage in these patient groups. In this study, we investigated the value of cTnT value in diagnosing acute coronary syndrome in CKD patients with high clinical suspicion of acute coronary syndrome and without acute ST segment elevation on electrocardiogram. The aim was to prevent cardiac ischemia from being overlooked in CKD patients.

Coronary angiography revealed vessel occlusion in 192 patients, and the mortality rate after treatment decisions was 6.7%. The first measured troponin results showed a significant difference in patients who did not survive, indicating the prognostic value of troponin levels. Troponin values were compared with cardiovascular pathologies obtained by angiography, and elevated troponin levels strongly correlated with pathologic angiography results.

The conclusion highlighted that despite prognostic uncertainties, biomarkers used for acute myocardial infarction diagnosis in patients with renal insufficiency are reliable in those with renal dysfunction. Elevated cTnT levels in CKD patients are considered a clear marker of cardiac ischemia, emphasizing the need for careful consideration of troponin values in this population.

## 1. Introduction

Chronic kidney disease (CKD) is a progressive loss of renal function defined as kidney damage that persists for 3 months or longer or an estimated glomerular filtration rate (eGFR) of <60 mL/min per 1.73 square meters.^[1]^ Studies indicate that chronic kidney disease will become the fifth leading cause of death worldwide in the next decade, and among the leading causes of this dramatic rise are its effects on the cardiovascular system.^[2]^ It has been reported that cardiovascular mortality is 57% higher in people with a GFR of <60 mL/min per 1.73 m^2^ and 63% higher in the presence of microalbuminuria, while the likelihood of having a myocardial infarction is 33% with a GFR of <60 mL/min per 1.73 m^2^ and 48% with microalbuminuria.^[3–6]^

Troponin, one of the cardiac markers, is a specialized component of the contractile mechanism of skeletal and cardiac myocytes. Troponin proteins and calcium ions regulate and facilitate the interaction between actin and myosin filaments as part of the sliding filament mechanism of muscle contraction. It is essential evidence of myocardial necrosis in patients with clinical signs suggestive of acute myocardial infarction. In patients with acute chest pain, clinical evaluation, history reminiscent of acute coronary syndromes (ACS), high-risk patient history, 12-lead electrocardiography (ECG), and cardiac troponin (cTn) I T or high-sensitivity troponin have diagnostic value. They are widely used in emergency department (ED) diagnostic and exclusion strategies for patients with chest pain, enabling clinicians to identify patients at risk.^[7,8]^ Although troponin values primarily suggest myocardial ischemia, they can also be elevated by other diseases such as myocarditis, Tako-tsubo cardiomyopathy, or shock. For the interpretation of the results, the clinic and history should be supported by the clinic.^[9]^

Cardiac troponins have high-reliability values, and there is no consensus on whether the diagnostic criteria for acute myocardial ischemia should differ for patients with and without renal dysfunction. Although it is often emphasized that the etiology of elevated troponin values in CKD is not fully explained, elevated cardiac troponin (cTnT) values are associated with increased subclinical cardiac damage in these patient groups.^[10]^

This study investigated troponin T (cTnT) value in diagnosing ACS in chronic renal failure patients (stage 3–5) with high clinical suspicion of ACS and without acute ST-segment elevation on 12-lead ECGs. We aimed to prevent cardiac ischemia from being overlooked in patients with CKD.

## 2. Methods

### 2.1. Study design

The study was performed at a university with the only angio unit in the region. Therefore, it serves a large population. The retrospective analysis identified 417 patients with a prediagnosis of ACS by analyzing clinical evaluation and patient history. Complete data sets and angiography results were reported, and 270 patients fulfilling the inclusion criteria were included.

Izmir Katip Celebi University Ethics Committee approved this retrospective study protocol (Dated: October 22, 2020 and numbered:1004).

### 2.2. Study data set

**Figure 1.**
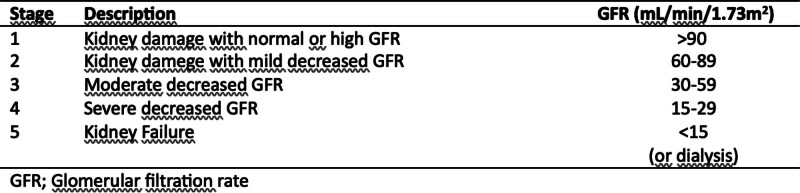
Kidney Disease Improving Global Outcome Classification.

CKD patients were selected according to the criteria in the 2004 Kidney Disease Improving Global Outcome (KDIGO) Guideline (Fig. 1). According to the KDIGO guideline, stage 3, stage 4, and stage 5 CKD patients were included in the study.^[11]^ Stages 1 to 2 were excluded from the study because they did not disrupt hemodynamics, glomerular filtration was maintained, and symptoms were faint or absent. In selected CKD patients, the absence of dialysis during and after the onset of chest pain was used as an acceptance criterion. The diagnosis of acute cardiac infarction without ST-segment elevation was based on the decision of at least 1 independent cardiologist, and patients who underwent angiography within the first 24 hours were included in the study. Angiography reports were considered for definitive diagnosis. In our study, the presence of stenosis above 50% in the main coronary structures accepted according to the 2021 ACC/AHA/SCAI guidelines was accepted as coronary occlusion.^[12]^

Patient selection was based on the absence of a history of major surgery or trauma in the previous 4 weeks, pregnancy, intravenous drug abuse, and anemia (hemoglobin < 10 g/dL). Other diagnoses mimicking cardiac syndromes (aortic dissection/aneurysm, pneumothorax, pulmonary embolism, etc) were excluded. The patients’ first referral center was considered the university ED, and patients referred from an external center or treatment initiated were excluded from the study.

### 2.3. Clinical data set

**Figure 2.**
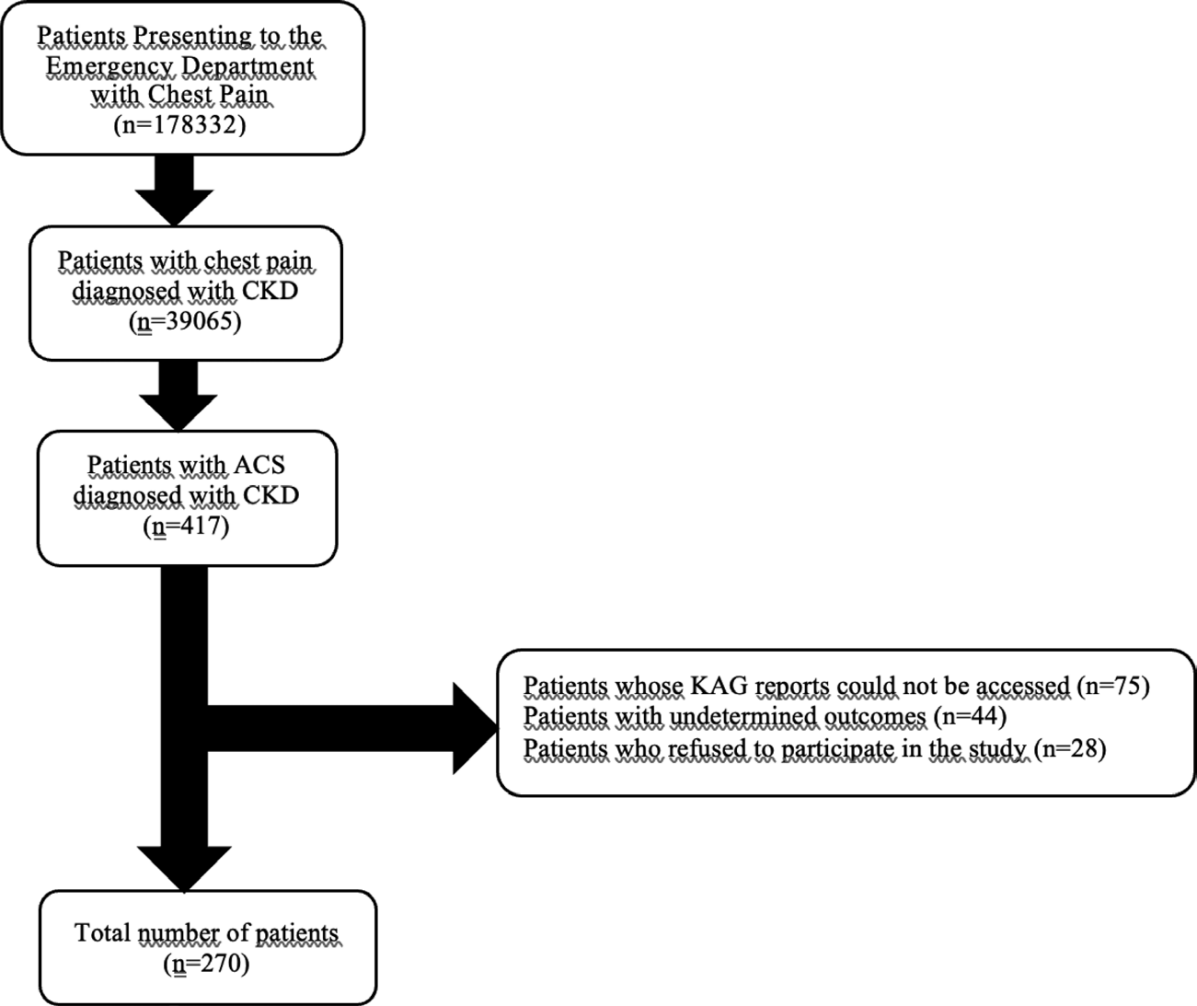
Consult diagram.

Patients admitted to the ED between 2015 and 2020 were analyzed from the hospital registration system. A clinical decision written by at least 1 emergency medicine physician and 1 cardiologist, with clinical data suggestive of an ACS, was considered decisive for prediagnosis. 12-lead ECG findings of ST-segment elevation pathologies were excluded. For the study, admissions made within the first 2 hours after the onset of pain and cardiac markers obtained within the first 6 hours after the onset of pain were recorded on patient forms (Fig. 2).

### 2.4. Blood sampling and laboratory methods

Troponin levels were analyzed with Roche Diagnostics Troponin T kit (TROPONIN T HSST (STAT) ELECSYS COBAS E 200) on Siemens Centaur Xp device, and the cutoff value range was 0 to 0.06 ng/mL. Creatine values were analyzed with an ABBOTT kit on the Abbott c16000 device, and the cutoff value range was 0.6 to 1.1 mg/dL.

### 2.5. Statistical analysis

Kruskal Wallis H Test was performed if the data were not normally distributed. The Dunn-Bonferroni test was used as a multiple comparison test if the result of the Analysis of Variance was significant. Mixed-order analysis of variance was used to compare the measurements between the groups. Bonferroni correction was applied to compare the main effects in mixed-order analysis of variance. Receiver operating characteristic curve analyses were used to evaluate the predictive performance of blood samples taken at admission (T1), blood samples taken at 6 hours (T2), and fold between 2 measurements (TF) parameters according to the outcome group. The relationships between numerical variables were evaluated with the Spearman correlation coefficient. The effect of T1, T2, TF, creatine kinase myocardial banding (CK-MB) 1, and CK-MB 2 variables on outcome was evaluated by logistic regression. *P* < .05 was considered statistically significant.

## 3. Results

The study included 270 patients. Of the patients, 100 were female and 170 were male. 102 (37.8%) patients were receiving routine dialysis. According to the KDIGO scale, 117 patients had stage 3, 62 had stage 2, and 91 had stage 5. Coronary angiography showed no coronary lesion in 78 patients, and vessel occlusion was identified in 192 patients. In 77 patients, medical treatment was decided, and coronary surgery was performed for 77 patients. The mortality rate was 6.7% after the treatments decided for the patients (Table 1).

**Table 1:** Descriptive statistics of patients.

Variables		Statistics
Age		66.12 ± 11.79 67 (32–109)
Gender	Female	100 (37)
	Male	170 (63)
Stage	3	117 (43.3)
	4	62 (23)
	5	91 (33.7)
ACS	USAP	134 (49.6)
	NSTEMI	136 (50.4)
Angiography result	Normal	78 (28.9)
	Single artery	77 (28.5)
	Double arteries	68 (25.2)
	Three arteries	47 (17.4)
Treatment	No treatment	7 (2.6)
	Medical treatment	77 (28.5)
	Interventional treatment	109 (40.4)
	Surgical treatment	77 (28.5)
Outcome	Discharged	252 (93.3)
	Ex	18 (6.7)

Usap = unstable angina pectoris, Nstemi = non ST segment elevation myocardial infarction.

**Figure 3.**
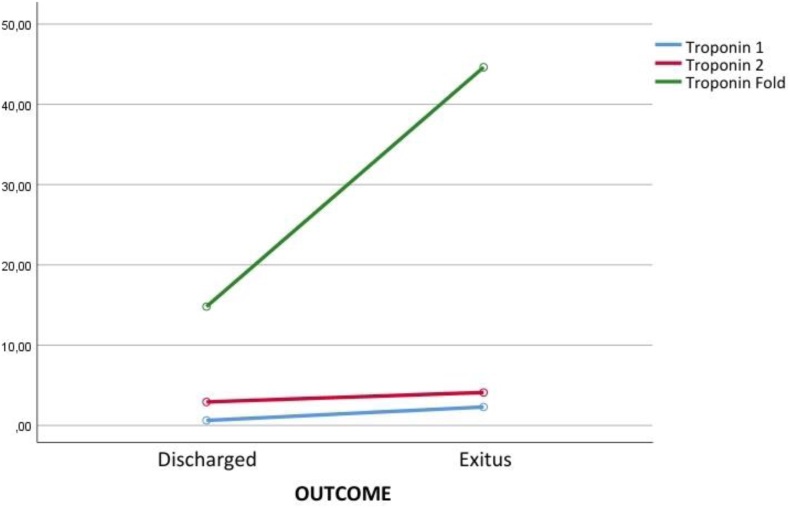
Troponin results according to outcome groups.

Troponin results were statistically significant between time and outcome groups (*P* = .001). There was a difference between T1, T2, and TF measurement times in discharged patients (*P* < .001). A significant difference was observed in the mean of the first measured Troponin results in patients with exitus outcome (Fig. 3).

The joint effect of time and outcome group on Ck-Mb findings was analyzed. A difference was observed between the outcome groups at the time of the first Ck-Mb measurement (*P* < .001). The first Ck-Mb value was statistically significant in patients with exitus outcome. Cardiac markers were compared in Table 2.

**Table 2:** Comparison of Troponin and Ck-Mb variables with outcome groups.

	Outcome		Test statistics†		
	Discharge	Exitus	*F*	*P*	*η* ^2^
*Troponin*					
T1	1.01 ± 3.4^x^	8.04 ± 12.86	**19.576**	**.001**	**0.070**
T2	2.84 ± 6.53^y^	4.1 ± 3.27	0.563	.454	0.002
TF	14.8 ± 65.7^z^	44.61 ± 168.7	2.531	.113	0.010
*Test statistics* *	***F* = *19.297; P* < .001; *η^2^* = 0.129**	*F* = *2.848; P* < .060*; η^2^* = 0.021			
	***F* = *2.878; P < *.001*; η^2^* = 0.047**				
*CK-MB*					
CK-MB 1	3.86 ± 6.11	23.22 ± 44.14	**22.588**	**.001**	**0.078**
CK-MB 2	7.59 ± 17.65	12.76 ± 12.11	1.487	.224	0.006
*Test statistics* ^¥^	***F* = *20.907; P* < .001*; η^2^* = 0.073**	*F* = *2.417; P* < .121*; η^2^* = 0.009			
	***F* = *7.207; P < *.008*; η^2^* = 0.026**				

F: mixed design ANOVA, effect size (*η*^2^). Descriptive statistics are given as mean ± standard deviation. Sections in bold are statistically significant (*P* < .05). There is no difference between the same letters. x, y, z; column-based lettering; a, b, c; row-based lettering.

T1 = Troponin 1, T2 = Troponin 2, TF = Troponin Fold.

* Intergroup comparison.

† Intergroup comparison.

In all analyzed stages of renal failure (stages 3–5), there was a difference between the first measured Troponin value (*P* < .001). Initial troponin values were associated with mortality (Table 3). However, no significant difference was seen for Ck-Mb values.

**Table 3:** Comparison of Troponin and Ck-Mb variables with stage groups in outcome groups.

		Stage			Test statistics†		
		Stage 3	Stage 4	Stage 5	F	*P*	η2
Discharged	*Troponin*						
	T1	0.63 ± 1.96^ax^	1.02 ± 2.67^ab^	1.52 ± 4.98 ^ab^	**3.253**	**.040**	**0.026**
	T2	2.62 ± 6.09^y^	2.14 ± 3.69	3.62 ± 8.37^y^	1.058	.349	0.009
	TF	12.48 ± 33.04^xy^	5.34 ± 6.69	24.57 ± 107.90^z^	1.540	.216	0.013
	Test statistics*	***F = 7.825; P < *.001*; η^2^ = *0.061**	*F = 2.028; P < *.134*; η^2^ = *0.017	***F = 9.919; P < *.001*; η^2^ = *0.076**			
		***F = 15.207; P < *.001*; η^2^ = *0.059**					
	*CK-MB*						
	CK-MB 1	3.5 ± 5.57	3.71 ± 3.3	4.44 ± 8.04	0.755	.471	0.006
	CK-MB 2	8.5 ± 20.43	8.15 ± 21.32	5.97 ± 8.46	0.522	.594	0.004
	Test statistics*	***F = 13.783; P < *.001*; η^2^ = *0.053**	***F = 4.755; P < *.030*; η^2^ = *0.019**	*F = 2.758; P < *.098*; η^2^ = *0.011			
		***F = 68.065; P < *.001*; η^2^ = *0.215**					
Exitus	*Troponin*						
	T1	7.03 ± 10.45	10.94 ± 18.21^x^	5.71 ± 7.17^x^	1.624	.230	0.178
	T2	3.13 ± 3.97	2.55 ± 2.89^y^	5.69 ± 2.64^y^	1.900	.184	0.202
	TF	8.61 ± 15.82	147.80 ± 319.89^xy^	2.62 ± 1.71^xy^	1.349	.289	0.152
	Test statistics*	*F = 3.081; P < *.078*; η^2^ = *0.306	***F = 6.724; P < *.009*; η^2^ = *0.490**	***F = 14.055; P < *.001*; η^2^ = *0.668**			
		*F = 2.186; P < *.160*; η^2^ = *0.127					
	**CK-MB**						
	CK-MB 1	30.8 ± 52.92	13.2 ± 24.94	27.6 ± 54.57	1.845	.192	0.197
	CK-MB 2	15.63 ± 21.52	7.3 ± 4.41	14.38 ± 7.07	0.695	.514	0.085
	Test statistics*	***F = 5.469; P < *.034*; η^2^ = *0.267**	*F = 0.518; P < *.483*; η^2^ = *0.033	*F = 2.161; P < *.162*; η^2^ = *0.126			
		***F = 23.102; P < *.001*; η^2^ = 0.606***					

F: mixed design ANOVA, effect size (*η*^2^). Descriptive statistics are given as mean ± standard deviation. Sections in bold are statistically significant (*P* < .05). There is no difference between the same letters. x, y, z; column-based lettering; a, b, c; row-based lettering.

T1 = Troponin 1, T2 = Troponin 2, TF = Troponin Fold.

* Intergroup comparison.

† Intergroup comparison.

**Figure 4.**
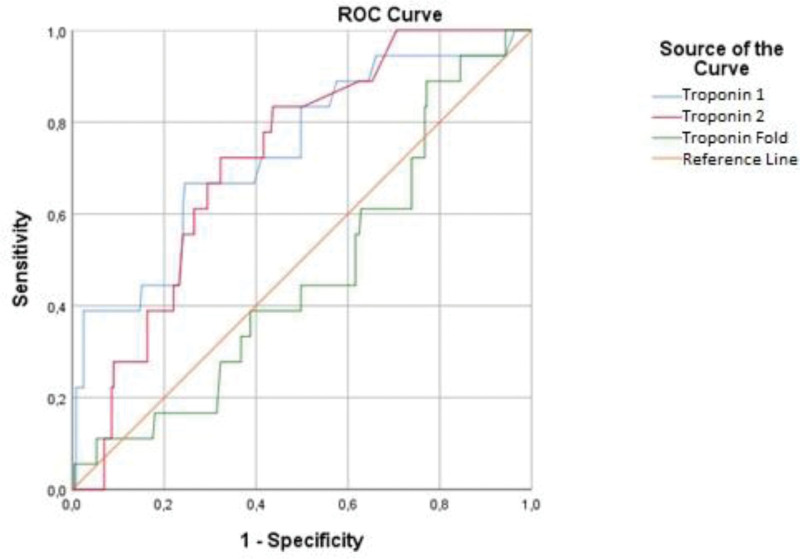
ROC curves for T1, T2, and TF measurement parameters.

The highest area under the curve cutoff value for the first analyzed Troponin was >0.6, sensitivity 83.33%, and specificity 57.54%. The optimum cutoff value for control Troponin was >1, sensitivity 71.14% and specificity 72.03%. The optimum cutoff value for Troponin Fold was ≤1.9423, sensitivity 55.56%, and specificity 61.63%. According to the cutoff values calculated for the markers, at least a 10-fold increase in troponin value compared to the cutoff value, at least 16.7-fold increase in the control cardiac value taken at 6 hours compared to the average cutoff value, and 1.94-fold increase in the fold in cardiac markers between the 2 values are significant for acute coronary event. The roc curve analysis of cardiac markers according to outcome groups is presented in Table 4 (Fig. 4).

**Table 4:** Cutoff scores, AUC value, sensitivity, selectivity and statistical significance by endpoint group with T1, T2, and TF measurements.

Test result variables	Cutoff	AUC	Std. error	*P*	Asymptotic 95% confidence interval		Sensitivity	Specificity
					Lower bound	Upper bound		
T1	>0.6	0.764	0.0553	**.001**	0.711	0.811	83.33	57.54
T2	>1	0.726	0.0499	**.001**	0.668	0.778	71.14	72.03
TF	≤1.942	0.531	0.0686	**.001**	0.469	0.592	55.56	61.63

AUC = area under the curve, CI = confidence interval, T1 = Troponin 1, T2 = Troponin 2, TF = Troponin Fold.

*Adjusted for age.

T1, T2, and TF values were compared with cardiovascular pathologies obtained by angiography. Marker values at least a 10-fold increase from normal limits or T1 > 0.6 ng/mL strongly correlated with pathologic angiography results. Similarly, a significant T2 > 1 ng/mL value and a 2-fold increase in mean TF collinear with cardiovascular occlusion. Marker values below significant troponin or increase coefficients are consistent with normal coronary anatomy.

Comparison of Troponin Variables with Angiography Outcome Groups is presented in Table 5.

**Table 5:** Comparison of Troponin variables with angiography result groups.

	Angiography result				Test statistic	*P*
	Normal	Single artery	Double arteries	Three arteries		
T1	0.2 ± 0.51^a^	1.3 ± 5.55^b^	1.82 ± 4.45^b^	4.03 ± 8.59^c^	38.933	**.001**
T2	0.98 ± 2.75^a^	3.14 ± 6.73^b^	3.3 ± 4.53^b^	5.26 ± 10.36^b^	28.756	**.001**
TF	6.02 ± 10.84^a^	12.98 ± 35.64^b^	33.12 ± 127.76^b^	17.12 ± 88.39^b^	8.637	**.035**

Numerical variables are given as mean ± standard deviation.

T1 = Troponin 1, T2 = Troponin 2, TF = Troponin Fold.

*One-way ANOVA.

†Kruskal–Wallis test.

The relationship between cardiac markers and outcome and mortality was analyzed. Each one-unit increase in troponin F value increases the probability of exitus 1.005-fold and for CK-MB 1, a one-unit increase increases the probability of exitus 1.269-fold. The Logistic Regression Model for Outcome is presented in Table 6.

**Table 6:** Logistic regression model for outcome.

	Regression coefficients						
	β	Standard error	Wald statistics	*P*	Exp (β)	95% C.I. for exp (β)	
						Lower limit	Upper limit
Constant	–3.571	0.446	64.007	.001	0.028		
T1	0.295	0.193	2.326	.127	1.343	0.919	1.962
T2	–0.202	0.150	1.810	.178	0.817	0.609	1.096
TF	0.005	0.002	6.546	**.011**	1.005	1.001	1.009
CK-MB 1	0.238	0.086	7.720	**.005**	1.269	1.073	1.501
CK-MB 2	0.002	0.024	0.011	.917	1.002	0.957	1.050

Variables included in the model: Troponin 1, Troponin 2, Troponin Fold, CK-MB 1, CK-MB 2.

Model statistics: Hosmer and Lemeshov test χ^2^ = 6.579; *P* = .583; Nagelkerke *R*^2^ = 0.216.

Elimination method: enter.

Β:coefficient of regression.

## 4. Discussion

Cardiovascular pathologies are more common in patients with chronic renal failure. CKD itself can be considered the equivalent of coronary artery disease, and people with early stages of CKD are more likely to die from CVDs. These patients are likelier to have silent ischemia or atypical presentations.^[13]^ However, electrolyte abnormalities left ventricular hypertrophy, subclinical myocyte damage from micro-infarctions, and concomitant congestive heart failure, which are commonly observed in CKD patients, are blamed for elevated cardiac troponins and laboratory values indicating cardiac ischemia are ignored.

A review of different publications may suggest that using troponin values for ACS is still controversial, and the prognostic significance of high troponin values remains unclear. However, both patient-based statistical studies and cardiac and renal function analyses suggest that high troponin values are strongly correlated with cardiac ischemia. In a study on rats with impaired renal function in which Vincent Fridén et al analyzed whether an enormously increased cTnT value was due to inadequate renal clearance, it was shown that potentially low cTnT levels would be primarily related to decreases in renal clearance, but that cTnT measured at high concentrations was due to myocardial infarction and should not be attributed to renal filtration deficiencies.^[14]^ As a result of his study on volunteers, Van der Linden stated that decreased renal clearance was not the leading cause of high troponin levels in patients with impaired GFR and declared that high cTnT values resulted from cardiac-induced release as a result of myocardial damage. The study demonstrated that impaired renal elimination was not the main factor behind elevated cardiac cTnT levels. It emphasized the importance of comprehensive diagnostic investigations in all patients with elevated cardiac troponin concentrations, regardless of their GFR.^[15]^ It is possible to see similar results in articles based on patient-based statistical data. In a meta-analysis of 23 articles on troponin levels and renal failure, SR Stacy et al concluded that high troponin values should be associated with cardiac infarction despite low sensitivity and specificity.^[16]^

The results of our study also support the current literature. It is methodologically simple and easy to use. Suppose patients have typical clinical markers indicating a cardiac ischemia or syndromes equivalent to cardiac pain. In that case, elevated cardiac markers should be considered as the presence of a cardiac fact and a coronary lesion if, when evaluated together with ECG findings, the cTnT value (T1), which is considered positive considering the range considered normal, is increased at least 10-fold, or if the value (T2), measured at a time interval exceeding 6 hours after the onset of symptoms, is increased approximately 17-fold. Despite lower sensitivity and specificity, an average 2-fold increase in the time interval between T1 and T2 compared to the first measured value (TF) should be interpreted in favor of myocardial ischemia.

Patients presenting with chest pain or angina equivalents in patients with CKD, that is, patients with ACS, were included in our study. However, it should not be overlooked that troponin elevation may be secondary to many pathological conditions such as CKD, infections, sepsis and anemia. Therefore, anamnesis, clinic, and ECG are very important.

We found high mortality rates in patients with high initial troponin values. Interpreting cTnT measurements at high reference values in favor of insufficient clearance in the renal clearance will lead to overlooking a subclinical myocardial injury and occlusion. When evaluated together with clinical findings, high cTnT values above the reference value, which in our study should be at least a 10-fold increase, would provide a decision in favor of the patient to consider cardiac damage.

## 5. Conclusions

Despite current controversies and prognostic uncertainties, biomarkers preferred for diagnosing acute myocardial infarction in patients with renal insufficiency are also highly reliable markers in patients with renal dysfunction.^[17]^ High cTnT levels cannot be explained primarily and solely by renal clearance. In patients with reduced GFR, high cTnT levels are a clear marker of cardiac ischemia.

## Acknowledgments

The authors are grateful to everyone who contributed to this study and provided moral support.

## Author contributions

**Conceptualization:** Ecem Ermete Güler, Umut Payza, Ahmet Kayali, Süleyman Kirik.

**Data curation:** Ecem Ermete Güler, Umut Payza, Ahmet Kayali.

**Formal analysis:** Ecem Ermete Güler, Umut Payza, Ahmet Kayali, Efe Kanter.

**Funding acquisition:** Umut Payza.

**Investigation:** Ecem Ermete Güler.

**Methodology:** Ecem Ermete Güler, Ahmet Kayali, Süleyman Kirik, Efe Kanter.

**Project administration:** Ecem Ermete Güler, Umut Payza, Ahmet Kayali.

**Resources:** Ecem Ermete Güler, Umut Payza, Ahmet Kayali, Süleyman Kirik, Efe Kanter.

**Software:** Ahmet Kayali, Süleyman Kirik, Efe Kanter.

**Supervision:** Ecem Ermete Güler, Umut Payza, Ahmet Kayali, Süleyman Kirik, Efe Kanter.

**Validation:** Ecem Ermete Güler, Umut Payza, Ahmet Kayali, Süleyman Kirik, Efe Kanter.

**Visualization:** Ecem Ermete Güler, Umut Payza, Ahmet Kayali, Süleyman Kirik, Efe Kanter.

**Writing – original draft:** Ecem Ermete Güler, Umut Payza, Ahmet Kayali, Süleyman Kirik, Efe Kanter.

**Writing – review & editing:** Ecem Ermete Güler, Umut Payza, Ahmet Kayali, Süleyman Kirik, Efe Kanter.
